# Editors’ Book Table

**Published:** 1874-01

**Authors:** 


### Editors' Book Table.

[NOTE. — All works reviewed in the columns of the CHICAGO MEDICAL JOURNAL may be found in the extensive stock of W. B. KEEN, COOKE & CO., whose catalogue of Medical Books will be sent to any address upon request.]

*The Physicians' Visiting List for* 1874.

The name of "Lindsay & Blakiston" has become so familiar to physicians, who have carried it about in their pockets, stamped upon the "tuck" of their "Visiting List," that it has become identified with their financial as well as their literary interests. The age of this little pocket companion, twenty-three years, is the best commentary upon its value.

To the vast majority of medical men the "Visiting List" would be an economical investment at ten times its cost. H.

*The Physicians' Hand Book for* 1874. By WILLIAM E. ELMER, M.D., and ALBERT D. ELMER, M.D., New York. W. A. Townsend, publisher. 1874.

An excellent manual for recording and preserving memoranda not only of visits paid and to be paid, but also of clinical observation and experience, with much valuable matter beside, in the shape of a list of poisons and antidotes, designed, as far as possible, to provide the practitioner against every emergency. It has acquired large popularity, and has become indispensable to many physicians. H.

*A Manual of Midwifery, including the Pathology of Pregnancy and the Puerperal State.* By Dr. KARL SCHRŒDER, Professor of Midwifery and Director of the Lying-in Institution in the University of Erlangen. Translated into English from the third German edition, by CHARLES H. CARTER, B.A., M.D., B.S., London, etc., etc. With twenty-six engravings on wood. New York: D. Appleton & Company. 1873.

The Messrs. Appleton are entitled to the thanks of the profession for this, the first American, edition of Schrœder's Manual, the best upon the subject with which we are acquainted. It is the result not only of exhaustive and laborious research into the literature of gynæcology, but of thorough and accurate practical familiarity with the subject. The style of the book is one of its chief merits—it is concise, clear, and emphatic, without becoming dogmatical. Free from useless verbiage, the author says more in three hundred and eighty pages, and says it better, than is usually expressed in twice the number. His views and opinions seem to be "up to the times," and in harmony with the results of the most recent investigators. The author is to be commended most highly for appending his citations of evidence from authorities, in the form of simple references as foot notes, instead of resorting to the trick of "padding" by voluminous quotations, a modest octavo into a ponderous tome. H.

*The Student's Guide to Medical Diagnosis.* By SAMUEL FENWICK, M.D., F.R.C.P., Assistant Physician to the London Hospital. From the third Revised and Enlarged English Edition. With eighty-four illustrations on wood. Philadelphia: Henry C. Lea. 1873. Pp. 328.

This book, we are informed, grew out of a want which the writer experienced whilst teaching, clinically, in the hospital with which he is connected. Hence it is thoroughly, and we might almost say, intensely, practical throughout. Most works on the subject devote a large portion of their space to discussions of rare and "interesting" cases—such as are exceedingly exceptional in general practice. Its constant reference to physiology and pathological histology, as now taught, are peculiarly refreshing to those of us who believe that both these departments of knowledge are indispensable and invaluable to the physician of the present generation.

By the aid of its numerous *genuine* illustrations, the text is rendered comprehensible, almost at a glance.

The order of exposition of the varied topics coming under review is admirable, and while we regret the necessity felt by the able author of keeping the volume within such modest limits, it is due to him to state that we have rarely seen so much positive instruction concentrated in clear, as well as concise, form in so small a book. It is most cordially and conscientiously recommended to all medical students, whether they have or have not as yet gained their M.D. A.

*The Preventive Treatment of Calculous Disease, and the Use of Solvent Remedies.* By Sir HENRY THOMPSON, F. R. C. S., Surgeon Extraordinary to H. M. the King of the Belgians, Surgeon and Professor of Clinical Surgery to University College Hospital. Philadelphia: Lindsay & Blakiston. 1873.

This little work of seventy-two pages consists of two lectures delivered in answer to numerous inquiries addressed to their distinguished author upon the subjects designated. The reputation of the author as an authority upon this and kindred subjects is exceeded by that of no one living, hence such a production of his pen cannot fail to possess great practical value. He reprobates strongly the too common superficial practice of removing symptoms, *i. e.*, urinary deposits, under the presumption that thereby the morbid condition of which this is a result only, is removed, and urges the necessity of radical constitutional modifications.

Sir Henry, as might be expected, is a warm advocate for the method of removing calculi by the operation of lithotripsy, that being the special proceeding upon whose successful application so much of his well merited fame is based. He is not very enthusiastic about the practicability of removing these concretions by solvents nor by electrolysis. H.

*Lacerations of the Female Perineum and Vesico-Vaginal Fistula—their History and Treatment.* By D. HAYES AGNEW, M.D., Professor of Surgery in the University of Pennsylvania. With numerous illustrations. Philadelphia: Lindsay & Blakiston. 1873.

Many valuable contributions to medical literature are practically lost to the majority of the profession, from having been published seriatim in journals and subjected to the accidents necessarily pertaining to a file of periodicals. The labor involved in their examination often involves such a serious waste of time, as to result in their being consigned to unmerited oblivion.

The collection and publication in book form of such papers is a work deserving the highest commendation, and especially so, when they possess so much real merit as these of Dr. Agnew. The little volume is a condensed summary of all that is worth knowing, to the general practitioner, concerning the subjects of which it treats, and we know of few more valuable little books. H.

*Annual Report of the Surgeon General United States Army*, 1873.

The financial statement of the "Report" shows that the expenditures of the medical department of the army have been made with rigid economy, the average expense per capita of cases being exceedingly small, *i. e.*, less than five dollars.

The portion devoted to the "Health of the Army during the fiscal year, ending June 30, 1873," presents some interesting information. From this it appears that the ratio of sickness and of casualties was greater among the white than among the colored troops. This difference in the ratio is still more marked, in the same direction, when we read the number of "disability discharges."

The "Army Medical Museum" has now become an object of interest to the medical profession throughout the country, and it is gratifying to perceive the evidences of its continued increase in the number and value of its specimens.

Of the "Medical and Surgical History of the War," five thousand copies have been distributed. The eagerness with which they have been sought for by the profession generally, constitutes the best testimonial of their value.

We take the liberty of quoting the language of the Surgeon General from page eleven of the "Report," upon a subject which we think demands the sympathy and hearty co-operation of every medical man in the country who has the honor of his profession at heart. He says: "I am compelled to again repeat the statement made in previous reports, that very serious and increasing injury has resulted to the service from the continued prohibition of appointments and promotions in the medical corps. The inducements of pay and rank as at present established, are not sufficient to make the service attractive or remunerative to physicians already engaged in practice, and though, through the prerequisite examination of candidates, it has heretofore been found possible to secure a high grade of talent and qualification, it is upon the younger portion of the profession, the recent graduates, that we must depend in filling up existing vacancies. As a large proportion of applicants fail to pass satisfactory examinations (which require in each case from three to six days,) it will be the work of several years to restore the corps to the necessary standard of numbers, as provided for in the Act of Congress approved July 28, 1866.

"Although ambition to pass the Army Medical Board brings forward many of the most prominent graduates of medical colleges, additional inducements of rank, pay and promotion are becoming more and more necessary, not only to make the number of candidates equal to the needs of the service, but to retain the most desirable of them in the service under their frequent inducements to accept advantageous offers in civil life.

"The action of the American Medical Association, representing the medical profession of the country, regarding the unequal position of medical officers of the army, as compared to that of other staff corps, is based upon actual investigation of the subject, and presents to Congress all the facts in the case. I can only urge most earnestly upon your attention the pressing and absolute necessity for such legislation as will secure to our officers and soldiers the efficient and reliable attendance in wounds and sickness which the Government should provide, and will make a position in the medical corps of the army, now as formerly, an object of ambition to the best educated and best qualified young men in the profession."

There are "at present sixty-four (64) vacancies in the corps, viz., two (2) assistant medical purveyors, five (5) surgeons, fifty-six (56) assistant surgeons, and one (1) medical storekeeper."

We have quoted the above at length, as it expresses much more clearly than we could do, the great injustice done to a large number of the most accomplished and deserving members of our profession, by reason of the niggardly policy of Congress, in restricting the appropriation for the pay of the department.

We have had the opportunity, to some extent, of observing personally some of the evils of which the Surgeon General complains, and most heartily endorse every word of the above quotation. We ask, therefore, every one of our readers to use his influence directly and personally with his Representative in Congress to induce him to remedy this great injustice. In thus aiding the Army Medical Corps in securing their rights, he will be rendering most efficient service to his own profession, and thus help himself. We believe if every one of our readers will make it his business to comply with our request, the object will be accomplished. H.

*Memorial of the American Medical Association with regard to the Rank of the Medical Corps of the United States Army.*

The pamphlet with the above title embodies one of the most meritorious works of the American Medical Association, and if the object therein set forth be accomplished, the Association will not have lived in vain. The idea of republishing in pamphlet form their action upon this matter was good, as it will thereby reach many who would never have found it amidst the dry details of the ordinary "Transactions."

Editorial comment would be superfluous, inasmuch as we have already expressed ourselves very decidedly upon the injustice done by Congress to the Army Medical Corps. This Memorial should have a much wider circulation than the Transactions will probably have. H.

*A Graceful Tribute.*

We have received from Mr. Henry C. Lea, of Philadelphia, the American publisher, a "preface" for the American edition of Taylor's Principles and Practice of Medical Jurisprudence, which was accidentally delayed until after the publication of the work. We have already noticed this great work editorially, but nevertheless feel that so graceful a compliment to one of our own country and profession deserves special notice and republication in full, viz:

"In the year 1825, when a medical student at Guy's Hospital, I accidentally met with a work entitled 'Elements of Medical Jurisprudence, by Theodoric Romeyn Beck, 2nd ed., with notes by W. Dunlop." I have that volume now before me, and I well remember the deep interest with which I read and studied its contents. I was then a student of two years standing, and the subject, from the lucid manner in which it was treated by the author, fixed my attention, and induced me for the time to put aside anatomy and physiology for the sake of this new branch of medical science. No lectures on the subject were then delivered in England, and Dr. Beck's work was the leading authority for lawyers and medical men.

"I believe that the sight of this book was the turning point of my selection of Medical Jurisprudence as a special object of study and practice. After six years of medical study in the schools of England, France and Italy, I received the appointment of Lecturer on Medical Jurisprudence from the Treasurer and Governors of Guy's Hospital, and from March, 1831, I have delivered an annual course of lectures on this subject in the medical schools connected with the hospital.

"In looking back over the forty-eight years since I received my first lesson in Medical Jurisprudence from the work of the late Dr. T. R. Beck, it is a great gratification to me to feel that I have been able to contribute to the literature of the science, and that my contributions have been so highly appreciated in the country which gave birth to Dr. Beck. For many years his was the only work on the subject in England and America.

"It is a satisfaction to me to know that I have been able to make some return to the country of his birth for the impulse which the study of his excellent treatise gave to me in my early days as a medical student. Dr. T. R. Beck has passed away, but his work, which had reached a tenth edition in 1851, will carry down his name to future years, as one of the most erudite and distinguished writers on Medical Jurisprudence."

### BOOKS RECEIVED.

*The Theory and Practice of Medicine.* ROBERTS. Lindsay & Blakiston.

*An Essay on the Principles of Mental Hygiene.* By D. A. GORTON, M.D. J. B. Lippincott & Co.

*Mind and Body.* By Sir BENJ. BRODIE. William Wood & Co., New York.

*Bowman's Practical Chemistry.* Henry C. Lea, Philadelphia.

*Lectures on Diseases and Injuries of the Ear.* By W. B. DALBY, F.R.C.S.E. Philadelphia: Lindsay & Blakiston.

### JOURNALS RECEIVED.

*The Practitioner*—October and November, 1873. McMillan & Co.

*The American Practitioner.* Louisville: J. P. Morton & Co.

*The Medical Record*—November and December, 1873. Wm. Wood & Co., New York.

*The New York Medical Journal*—November, 1873. D. Appleton & Co., New York.

*Philadelphia Medical Times*—Oct. 25, Nov. 1, 8, 15, 22, 29. J. B. Lippincott & Co.

*The Obstetrical Journal of Great Britain and Ireland*—October and November, 1873. Henry C. Lea, Philadelphia.

*The Medical and Surgical Reporter*, Nos. 17, 19, 20, 21, 22, 23. S. W. Butler, M.D., Philadelphia.

*The American Journal of the Medical Sciences*—October.

*The London Medical Record.* G. P. Putnam & Sons, New York.

*The London Lancet*—Oct. and Nov., 1873. William C. Herald.

*The Southern Medical Record*—Oct. and Nov., 1873. Atlanta, Ga.

*The Canada Medical Record.* Montreal, Sept., 1873.

*The Richmond and Louisville Medical and Surgical Journal.* E. S. Gaillard, M.D. Oct. and Nov., 1873.

*The Druggists' Circular*, New York, Nov. and Dec., 1873. Hagerty Bros. & Co.

*North-Western Medical and Surgical Journal*, St. Paul, Nov. and Dec., 1873.

*The Medical News and Library*—Nov. and Dec., 1873. Philadelphia: Henry C. Lea.

*Atlanta Medical and Surgical Journal*—Sept. and Oct., 1873.

*St. Louis Medical and Surgical Journal*—Nov., 1873.

*The Indiana Journal of Medicine*—Nov., 1873.

*The Ohio Medical and Surgical Reporter*—Nov., 1873.

*The Western Lancet*, San Francisco, Oct., 1873.

*Boston Journal of Chemistry*—Nov. and Dec., 1873.

*Pacific Medical and Surgical Journal*—Nov., 1873.

*The Nashville Journal of Medicine and Surgery*—Nov., 1873.

*The Detroit Review of Medicine and Pharmacy*—Nov. and Dec., 1873.

*The Kansas City Medical Journal*—October, 1873.

*The Eclectic Medical Journal*—Nov. and Dec., 1873.

*The Cincinnati Lancet and Observer*—Nov., 1873.

*The Canada Medical and Surgical Journal.*

*The Clinic*—Nos. 16, 17, 18, 20, 21, 22.

*Le Progres Medical*, Bonneville, Paris. No. 11, 13, 14, 15, 16, 17, 18, 19, 20.

*The Dental Cosmos*—Nov., 1873.

*Buffalo Medical and Surgical Journal.*

*The Medical Eclectic.*

*The Medical Examiner*, Chicago, Nov. 1 and 15, 1873.

*The Pharmacist*, Chicago, Nov., 1873.

*The Medical Herald*, Leavenworth, Kansas, Nov. 1, 1873.

PAMPHLETS RECEIVED.

*Medical Society of New Jersey.* Transactions. 1873.

*American Otological Society.* Transactions. 1873.

*The Function of the Eustachian Tube.* By THOMAS F. RUMBOLD, M.D., St. Louis, Mo.

*On the Granular Cell found in Ovarian Fluid.* By THOMAS DRYSDALE, M.D., Philadelphia.

*Memorial of the American Medical Association* with regard to the Rank of the Medical Corps of the U. S. Army.

*Annual Report of the Surgeon General U. S. Army*, 1873.

*Proceedings of Medical Association of the State of Arkansas.*

*Fetal Physical Diagnosis.* By FRANK C. WILSON, M.D., Visiting Physician to the St. Louis City Hospital.

*Smithsonian Miscellaneous Collection.* No. 266, Toner Lectures. Lecture 1.—On the Structure of Cancerous Tumors, and the mode in which adjacent parts are invaded. By J. J. WOODWARD, Assistant Surgeon U. S. A.

*Mortality Experience of American Missionaries.* NATHAN WILEY.

*White Sarcomatous Intra Ocular Tumor. Intra Ocular Tumor, White Tusiformed Cell Sarcoma, Enucleation. Hespes Zoster Ophthalmicus. Traumatic Rupture of the Choroid.* Papers by B. JOY JEFFRIES, Boston, Mass.

*Description of New Instruments for making Examinations of the Cavities of the Nose, Throat and Ear.* THOMAS F. RUMBOLD, M.D., St. Louis, Mo.

*Evidences of Life in the Newly Delivered Child.* By WILLIAM B. ATKINSON, M.D., Philadelphia.

*Expert Testimony.* By THAD. M. STEVENS, M.D., Indianapolis.

*Du Traitement des Retrecissements de l'Urethre par la dilatation progressive. Travail Couronnee par la Commission du Prix Civiale pour l'Anree.* 1872. Par T. B. CURTIS, Boston, Mass.

